# Enhanced Ti_0.84_Ta_0.16_N diffusion barriers, grown by a hybrid sputtering technique with no substrate heating, between Si(001) wafers and Cu overlayers

**DOI:** 10.1038/s41598-018-23782-9

**Published:** 2018-03-29

**Authors:** Marlene Mühlbacher, Grzegorz Greczynski, Bernhard Sartory, Nina Schalk, Jun Lu, Ivan Petrov, J. E. Greene, Lars Hultman, Christian Mitterer

**Affiliations:** 1 Department of Physical Metallurgy and Materials Testing, Montanuniversität Leoben, Franz-Josef-Strasse 18, A-8700 Leoben, Austria; 2 Thin Film Physics Division, Department of Physics, Chemistry, and Biology (IFM), Linköping University, S-581 83 Linköping, Sweden; 3 Materials Center Leoben Forschung GmbH, Roseggerstrasse 12, A-8700 Leoben, Austria; 4 Department of Materials Science, Physics, and the Frederick Seitz Materials Research Laboratory, University of Illinois, Urbana, Illinois 61801 USA; 5 Present Address: Department of Materials Physics, Montanuniversität Leoben, Franz-Josef-Strasse 18, A-8700 Leoben, Austria

## Abstract

We compare the performance of conventional DC magnetron sputter-deposited (DCMS) TiN diffusion barriers between Cu overlayers and Si(001) substrates with Ti_0.84_Ta_0.16_N barriers grown by hybrid DCMS/high-power impulse magnetron sputtering (HiPIMS) with substrate bias synchronized to the metal-rich portion of each pulse. DCMS power is applied to a Ti target, and HiPIMS applied to Ta. No external substrate heating is used in either the DCMS or hybrid DCMS/HiPIMS process in order to meet future industrial thermal-budget requirements. Barrier efficiency in inhibiting Cu diffusion into Si(001) while annealing for 1 hour at temperatures between 700 and 900 °C is investigated using scanning electron microscopy, X-ray diffraction, four-point-probe sheet resistance measurements, transmission electron microscopy, and energy-dispersive X-ray spectroscopy profiling. Ti_0.84_Ta_0.16_N barriers are shown to prevent large-scale Cu diffusion at temperatures up to 900 °C, while conventional TiN barriers fail at ≤700 °C. The improved performance of the Ti_0.84_Ta_0.16_N barrier is due to film densification resulting from HiPIMS pulsed irradiation of the growing film with synchronized Ta ions. The heavy ion bombardment dynamically enhances near-surface atomic mixing during barrier-layer deposition.

## Introduction

Diffusion barriers are vital components in integrated circuits (ICs), designed to impede interdiffusion between Cu metallization and doped Si layers^1,2^. Barrier failure leading to in-diffusion of Cu results in the formation of Cu silicides, which severely impair device performance and lifetime^1,3,4^. Since diffusion is a thermally-activated process, efficient diffusion barrier layers require a thermally-stable microstructure, with an electrical conductivity similar to that of Cu (1.74 µΩ-cm for bulk Cu, 2.0 µΩ-cm for a 1.5-µm-thick polycrystalline Cu film)^5,6^ in order to optimize device functionality^7^. Transition-metal nitrides are a diverse group of high-temperature ceramic materials, which can, in principle, fulfil these requirements. TiN, in particular, is a well-suited barrier material. It crystallizes in the B1 NaCl structure, which is stable up to the melting point, 2949 °C^8^. Structural and thermal stability is combined with a relatively low electrical resistivity of 13–25 µΩ-cm for epitaxial^9,10^ and polycrystalline^7,11^ layers.

However, the challenge in diffusion barrier synthesis lies in producing dense, low-defect TiN films, to avoid the presence of fast Cu-atom diffusion paths, at low temperatures (i.e., <100 °C) in order to meet increasingly stringent device thermal-budget requirements^12^. TiN layers sputter-deposited at low temperatures have a columnar morphology with continuous intercolumnar boundaries which can serve as direct diffusion pathways from the metallization layer to the underlying Si-layers^11,13^. We have previously shown that dense columnar TiN barriers can be grown by reactive sputter-deposition on Si(001) at *T*_*s*_ = 700 °C using an applied substrate bias of −100 V. The resulting barrier layer exhibits limited Cu diffusion after annealing at 900 °C for 1 h^14^. Similarly, the deposition of TiN by high-power impulse magnetron sputtering (HiPIMS) can result in a very dense microstructure if the deposition temperature is suitably high^15^. However, the International Technology Roadmap for Semiconductors (ITRS 2.0)^12^ shows that future thermal budgets prohibit the high-temperature growth of diffusion barriers.

A recent innovation in low-temperature thin film synthesis by Greczynski *et al*. is hybrid DC/high-power impulse magnetron sputtering (DCMS/HiPIMS), which has been shown to be capable of producing dense transition-metal nitride films without external substrate heating^16–18^. TiN densification is achieved by bombarding the growing film with short pulses of energetic metal ions sputter-ejected from a target operated in HiPIMS mode and accelerated to the growing film using a pulsed substrate bias applied in synchronous with the metal-ion rich portion of the HiPIMS pulses^17^. In principle, a certain degree of film densification can already be achieved by pulsed Ti^+^/Ti^2+^ metal-ion irradiation during Ti-DCMS/Ti-HiPIMS film deposition^19^. However, this is not nearly as effective as higher-mass Ta^+^/Ta^2+^ bombardment in densifying the growing film^16^. Therefore, we used the latter approach in which a Ta target is operated in HiPIMS mode and TiN is deposited continuously by DCMS to grow dense, dilute Ti_1−x_Ta_x_N alloy films (x = 0.16). Here, we evaluate whether this new hybrid deposition process can provide the missing link between the low-temperature processing needs of integrated circuit manufacturers and the requirement of a dense diffusion barrier microstructure.

In this article, we present the results of a comparative investigation of the diffusion barrier performance of DCMS TiN films and hybrid DCMS/HiPIMS Ti_0.84_Ta_0.16_N layers, both deposited without external substrate heating on Si(001) substrates with a native oxide layer. We employ a combination of scanning electron microscopy (SEM), X-ray diffraction (XRD), and four-point-probe sheet resistance (*R*_*S*_) measurements to study the topographical and microstructural evolution of the diffusion barriers as a function of annealing temperature *T*_*a*_ from 700 to 900 °C. Cu diffusion in barrier layers is investigated using cross-sectional transmission electron microscopy (XTEM) combined with energy-dispersive X-ray spectroscopy (EDX).

In the following sections, the DCMS TiN and DCMS/HiPIMS Ti_0.84_Ta_0.16_N films are referred to simply as TiN and Ti_0.84_Ta_0.16_N, respectively.

## Results and Discussion

### Topographical and microstructural evolution

**Figure 1:**
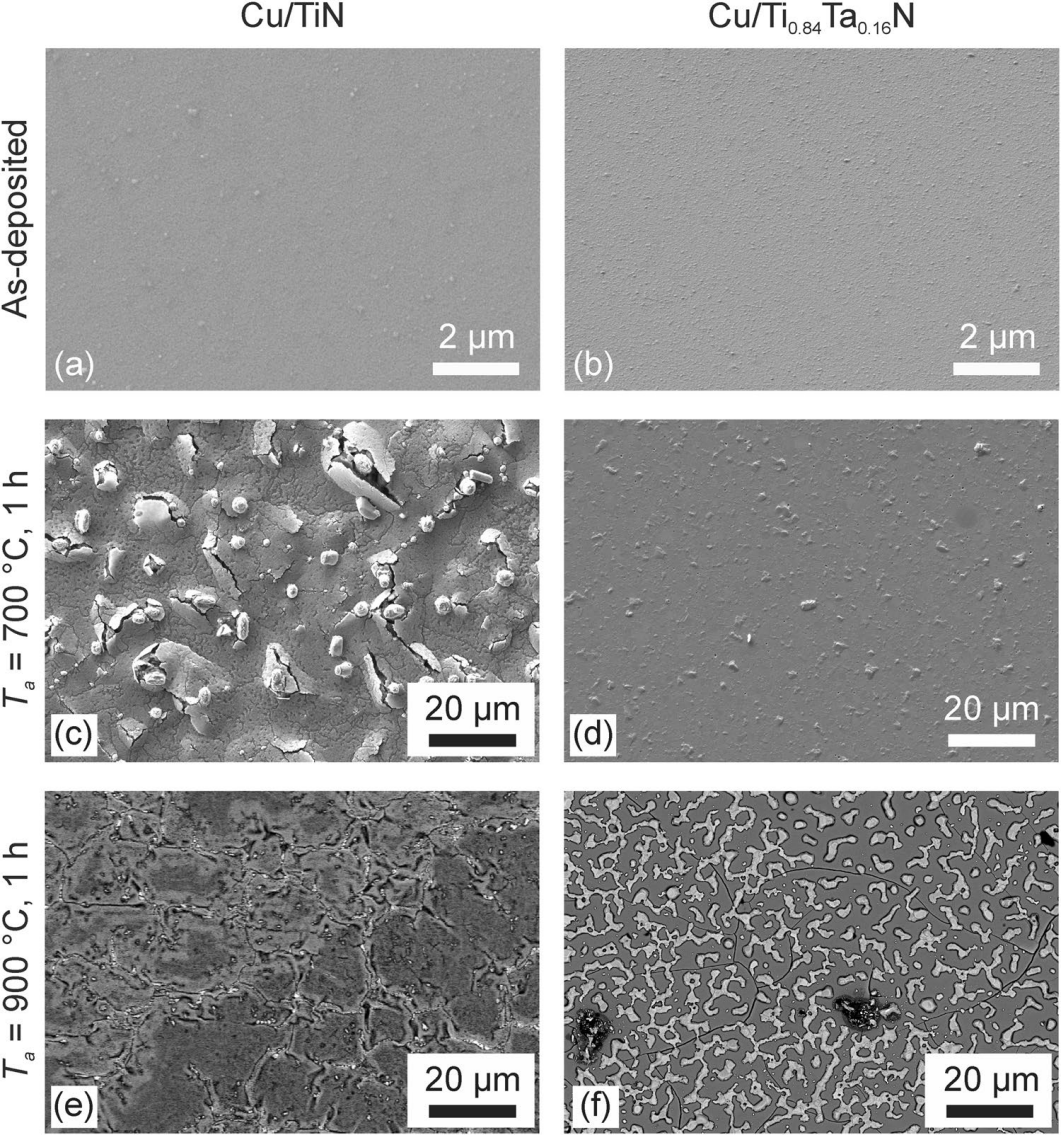
Plan-view SEM micrographs of as-deposited and annealed Cu/TiN and Cu/Ti_0.84_Ta_0.16_N bilayers deposited on Si(001) wafers. The images reveal topographical evolution as a function of *T*_*a*_. Images of as-deposited and 700 °C annealed samples are obtained with a secondary-electron detector; images of the 900 °C annealed samples with a backscattered-electron detector.

Figure 1 displays typical plan-view SEM images of Cu/TiN and Cu/Ti_0.84_Ta_0.16_N bilayers as-deposited (Fig. 1(a,b)) and after 1-h annealing at 700 (Fig. 1(c,d)) and 900 °C (Fig. 1(e,f)). The topography of both as-deposited samples is similar and reveals continuous Cu overlayers. Average roughnesses *R*_*a*_, obtained using white light interferometry, are 63.8 Å for Cu/TiN and 60.8 Å for Cu/Ti_0.84_Ta_0.16_N. The results are consistent with previous measurements showing that DCMS/HiPIMS TiTaN layers grown without substrate heating provide a smoother surface for Cu film growth than comparable DCMS TiN films^16^. Based upon XTEM analyses discussed in the next section, average Cu column diameters <*d>* are ~275 Å for Cu films on TiN and ~350 Å on Ti_0.84_Ta_0.16_N.

**Figure 2:**
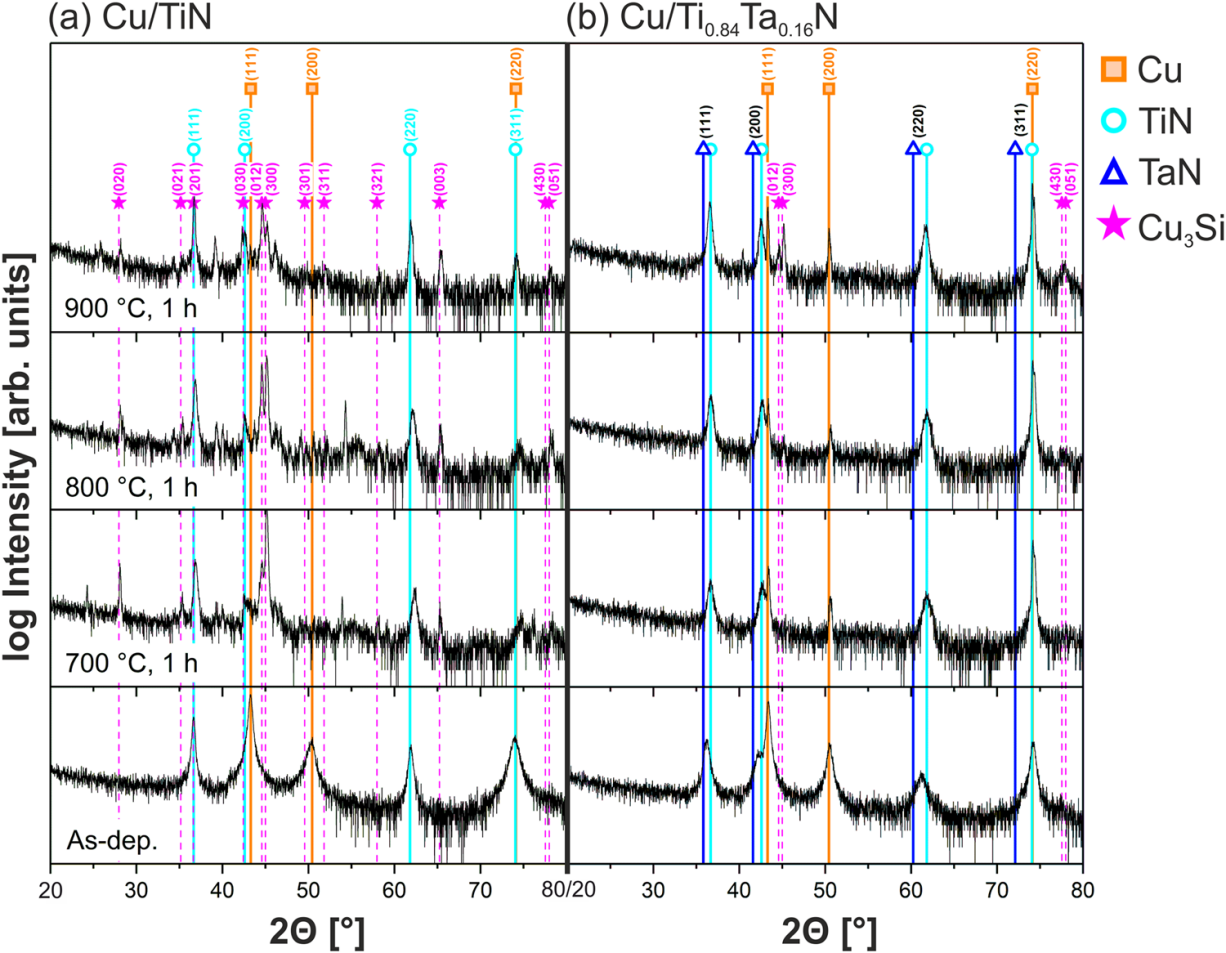
Evolution of XRD patterns, obtained at grazing incidence from (**a**) Cu/TiN and (**b**) Cu/Ti_0.84_Ta_0.16_N bilayers as-deposited and after 1-h annealing at 700, 800, and 900 °C on Si(001) substrates.

After 1-h annealing at 700 °C, Cu films grown on TiN exhibit cracks due to hillock penetration from underlying layers (Fig. 1(c)). In contrast, Cu layers deposited on Ti_0.84_Ta_0.16_N are continuous following 700 °C annealing (Fig. 1(d)) and the average column diameter has increased to ~5000 Å as determined by XTEM, with some grains visible in Fig. 1(d) in the µm-range, as previously reported for annealed Cu thin films^20–22^. A visual inspection with an optical microscope shows that the Cu overlayer on TiN, following 1-h 900 °C annealing, is no longer present. Instead, as shown in the backscattered-electron scanning micrograph in Fig. 1(e), the bare TiN surface exhibits a pronounced crack network, in which the cracks are partially filled with a bright-contrast phase. Since XRD scans presented in Fig. 2(a) show Cu_3_Si as the only reaction product in the sample volume after annealing, we conclude that this bright-contrast phase is Cu_3_Si (Fig. 1(e)). In contrast, the development of a bright Cu island network on the darker Ti_0.84_Ta_0.16_N surface is observable in Fig. 1(f) after annealing Cu/Ti_0.84_Ta_0.16_N for 1 h at 900 °C. This island network is the result of a solid-state dewetting process driven by the minimization of surface and interface energies. Cu grains within the island network have average sizes of several µm. In addition, cracks and isolated defects with a darker contrast are evident in the 900 °C annealed Ti_0.84_Ta_0.16_N film (Fig. 1(f)).

Typical XRD grazing incidence scans from as-deposited and annealed Cu/TiN and Cu/Ti_0.84_Ta_0.16_N samples are shown in Fig. 2. The 2θ scan range (2θ = 20 to 80°) in Fig. 2 was chosen to show the most intense Cu and TiN peaks. (111), (200), and (220) Cu reflections^23^, and (111), (200), (220), and (311) TiN reflections^24^, are obtained from the as-deposited Cu/TiN sample (Fig. 2(a)), indicating the polycrystalline nature of both films. TiN reflections appear at diffraction angles in excellent agreement with the ICDD reference pattern, suggesting the absence of residual stresses in the TiN layer. The further evaluation of residual stresses based on the sin^2^Ψ method in Seemann-Bohlin geometry^25^ is described in more detail in the supplementary information. A sin^2^Ψ plot (Supplementary Fig. S1) shows that the TiN film is essentially stress-free in as-deposited condition with a tensile stress of only 0.03% of the Young’s modulus *E*.

After annealing the Cu/TiN bilayer at 700 °C, additional diffraction peaks appear at 2θ = 28.0, 35.2, 42.4, 44.6, 45.0, 58.0, and 65.2° arising from the appearance of the orthorhombic *η*″-Cu_3_Si phase^26^. This compound is often reported as a reaction product due to Cu and Si interdiffusion through barrier layers in Cu/barrier/Si(001) stacks^27^, and therefore serves as a qualitative benchmark in evaluating barrier performance^1,3,4,27^. Formation of Cu_3_Si occurs locally at the barrier/Si interface^1,28,29^, and the 150% volume expansion^30^ compared to Si results in the formation of hillocks as observed in Fig. 1(c). The sharp peak followed by the broad hump at 2θ = 54° visible in the 700 and 800 °C annealed samples in Fig. 2(a) is the result of anomalous (non-Bragg) scattering in grazing incidence geometry from the single-crystal Si(001) substrate^31^. Further unassigned peaks in Fig. 2(a) are likely related to a Cu-oxide or a Cu-Ti or Si-Ti intermetallic compound, but could not be identified unambiguously. Since we do not detect the presence of either of these phases in subsequent analyses, we conclude that their fraction is negligible.

After annealing at 900 °C, no Cu peaks are discernible in XRD scans of the Cu/TiN bilayer samples. This indicates that the entire Cu layer has been consumed in forming Cu_3_Si, consistent with the plan-view SEM image in Fig. 1(e) showing a bare TiN layer with cracks containing Cu_3_Si visible as the bright contrast phase.

XRD patterns from as-deposited Cu/Ti_0.84_Ta_0.16_N bilayers (see Fig. 2(b)) consist of (111), (200), and (220) Cu reflections together with (111), (200), and (220) Ti_0.84_Ta_0.16_N reflections. The Ti_0.84_Ta_0.16_N peaks are located at positions between the corresponding reflections of the binary nitrides TiN and TaN^32^, indicating the formation of a solid solution with a NaCl structure. In annealed Cu/Ti_0.84_Ta_0.16_N samples, the Cu peaks increase in intensity and decrease in width with increasing annealing temperature. The decrease in peak width is consistent with Cu grain growth from <*d>* ~350 Å in as-deposited layers to several µm after annealing at 900 °C, as observed in SEM and XTEM images. We never obtain TaN XRD peaks indicating that the Ti_0.84_Ta_0.16_N solid solution is stable up to annealing temperatures of at least 900 °C. However, at all annealing temperatures, the three XRD peaks assigned to the as-deposited TiTaN solid solution - (111) at 2*θ* = 36.2°, (220) at 42.1°, and (220) at 61.2° - exhibit a shift toward higher 2*θ* values indicating the plane spacing in the film-growth direction decreases due to the release of compressive stress during annealing. sin^2^Ψ plots (Supplementary Fig. S2) reveal a compressive residual stress on the order of 1.5% of the Young’s modulus *E* of the Ti_0.84_Ta_0.16_N layer in as-deposited state. Stress relaxation occurs upon annealing, with the 900 °C annealed Ti_0.84_Ta_0.16_N layer essentially stress-free with a tensile stress of 0.09% of *E*. Since no stress build-up is detected, we conclude that TaN is not precipitating out of the solid solution upon annealing.

The formation of Cu_3_Si in the Cu/Ti_0.84_Ta_0.16_N bilayer sample on Si(001) is not observed at 800 °C, which is a definite improvement compared to the Cu/TiN barriers, in which it occurs at annealing temperatures below 700 °C. Presumably, Cu diffusion originates preferentially at local microstructural defects in the barrier layers, allowing Cu to react with Si at the barrier/substrate interface. The reaction product Cu_3_Si in 900 °C annealed Cu/Ti_0.84_Ta_0.16_N bilayers is evident at what appears to be randomly-spaced sites with darker contrast, see Fig. 1(f), in the backscattered-electron SEM image.

**Figure 3:**
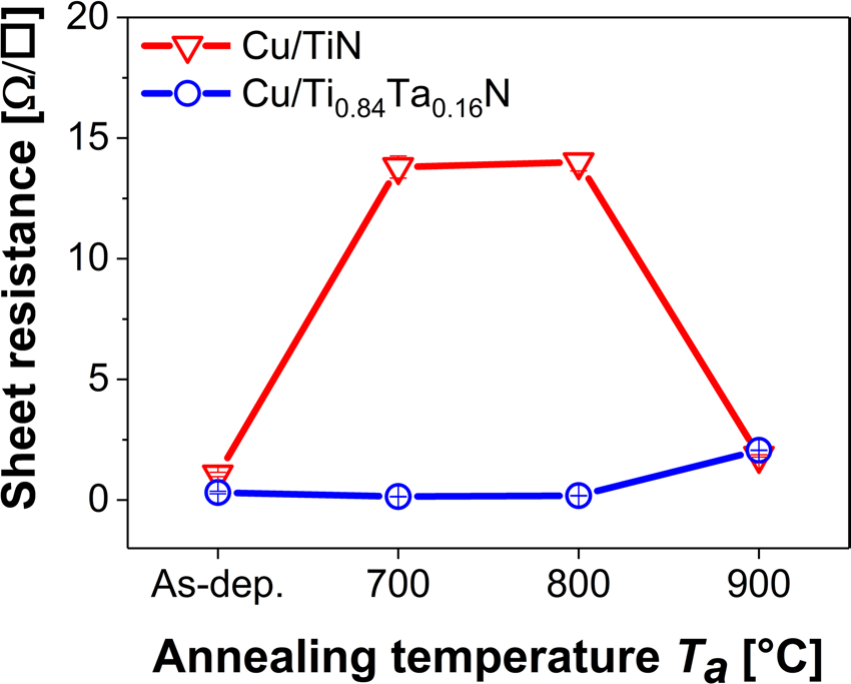
Evolution of the sheet resistances of Cu/TiN and Cu/Ti_0.84_Ta_0.16_N bilayers as a function of annealing temperature *T*_*a*_. The sheet resistances after 900 °C annealing correspond to the fully-reacted (Cu/TiN) and dewetted (Cu/Ti_0.84_Ta_0.16_N) bilayers.

The results of sample sheet resistance measurements presented in Fig. 3 are consistent with the SEM and XRD observations described above. The sheet resistance of as-deposited Cu/TiN bilayers, 1.06 ± 0.09 Ω/□, is considerably larger than that of the Cu/Ti_0.84_Ta_0.16_N bilayers, 0.31 ± 0.05 Ω/□, partly due to the smaller average column diameter <*d*> ~275 Å for Cu on TiN as compared to ~350 Å for Cu on Ti_0.84_Ta_0.16_N. Furthermore, Ti_0.84_Ta_0.16_N surfaces provide a smoother template^16^ leading to the growth of denser, larger-grained Cu films, whereas the rougher Cu/TiN interface is a source of additional electron scattering, leading to increased sheet resistance.

Annealing Cu/Ti_0.84_Ta_0.16_N bilayers for 1 h at 700 °C results in the sheet resistance decreasing to 0.14 ± 0.01 Ω/□, which is attributed to Cu grain growth to an average size of <*d>* ~5000 Å. After annealing at 800 °C, a small resistance increase to 0.18 ± 0.01 Ω/□ is observed, indicating the onset of the interdiffusion reaction. In contrast, the sheet resistance of Cu/TiN increases to 13.80 ± 0.46 Ω/□ after annealing at 700 and to 14.00 ± 0.36 Ω/□ at 800 °C, consistent with the appearance of the high-resistivity (55–60 µΩ-cm)^33,34^ Cu_3_Si phase in XRD scans (Fig. 2(a)) and the buckled structure in SEM images (Fig. 1(c,e)).

After annealing Cu/TiN at 900 °C, the entire Cu layer has been consumed in forming Cu_3_Si. From the Cu-Si phase diagram it is expected that local melting of the Cu_3_Si compound (melting point *T*_*m*_ = 859 °C) will occur during the 900 °C annealing treatment^35^. The sheet resistance corresponding to the fully-reacted TiN barrier layer is 1.83 ± 0.05 Ω/□. The sheet resistance of the 900 °C annealed, dewetted Ti_0.84_Ta_0.16_N barrier layer is 2.06 ± 0.01 Ω/□.

For comparison, sheet resistances are also measured for single-layer TiN and Ti_0.84_Ta_0.16_N samples deposited under the same conditions as the barrier layers in the corresponding bilayer samples. A detailed comparison is presented in Supplementary Table S3. In the as-deposited condition, the sheet resistance of the 2000-Å-thick TiN film is 42.22 ± 1.01 Ω/□, while the 1500-Å-thick Ti_0.84_Ta_0.16_N film yields a significantly lower value, 7.70 ± 0.15 Ω/□. The higher resistance of the as-deposited DCMS TiN film compared to the DCMS/HiPIMS Ti_0.84_Ta_0.16_N layer is due primarily to its lower density, ~65%^16^. After 1-h vacuum annealing at 900 °C, the sheet resistance drops to 2.59 ± 0.14 Ω/□ for single-layer TiN and 4.97 ± 0.49 Ω/□ for Ti_0.84_Ta_0.16_N because of defect annihilation. However, the resistance decrease is much more pronounced in TiN due to film densification upon annealing, whereas the Ti_0.84_Ta_0.16_N is already fully dense.

### Interdiffusion

**Figure 4:**
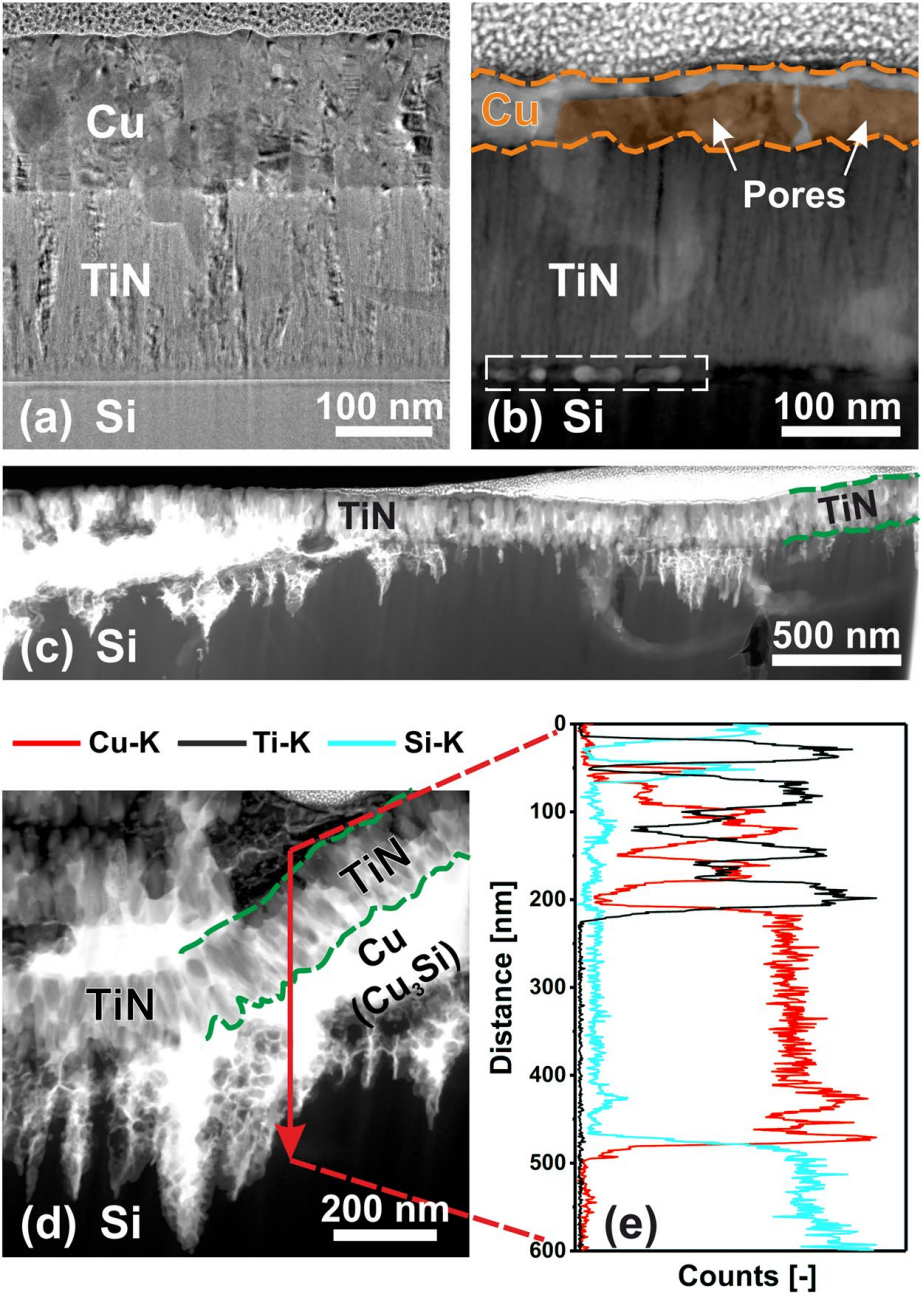
Cross-sectional transmission electron micrographs of a Cu/TiN bilayer grown on Si(001). (**a**) Bright-field XTEM image of the as-deposited bilayer, with the Cu layer schematically marked in orange, (**b**) *Z*-contrast STEM image of the bilayer after annealing at 700 °C, (**c**) *Z*-contrast STEM overview of the bilayer after annealing at 900 °C, and (**d**) higher-magnification STEM image of the bilayer annealed at 900 °C with (**e**) a corresponding qualitative EDX line scan acquired along the red arrow in (**d**).

To further investigate the effects of bilayer interdiffusion, bright-field XTEM and cross-sectional *Z*-contrast scanning transmission electron microscopy (STEM) images of as-deposited and annealed samples are acquired. Figure 4(a) is an XTEM image of an as-deposited Cu/TiN bilayer on Si(001). TiN grows with a columnar microstructure (average column diameter ~125 Å), exhibiting inter- and intracolumnar voids due to limited adatom mobility during film growth at low temperatures^36^. The Cu overlayer initially follows the columnar growth fashion (average column diameter <*d>* ~175 Å) from the TiN template, but gradually forms a more granular structure with larger columns (<*d>* ~275 Å) after approximately 500 Å.

Figure 4(b) is a *Z*-contrast STEM image from a 700 °C annealed Cu/TiN bilayer acquired in an area free of hillocks. The large-scale image contrast is due to differences in atomic mass. Thus, the Cu layer (m_Cu_ = 63.5 amu) appears brighter than the TiN (m_Ti_ = 47.9 amu, m_N_ = 14.0 amu) layer. The Cu layer is thinner after annealing than in the as-deposited case, indicating that a significant amount of Cu has already been consumed to form Cu_3_Si, in agreement with the SEM (Fig. 1(c)) and XRD (Fig. 2(a)) results. Even within the TiN layer, the brighter contrast indicates the presence of diffused Cu.

Contrast difference is also noticeable within the Cu layer itself. During thinning of the XTEM lamella in the focused-ion-beam (FIB) unit, a nano-sized pore was exposed at the Cu/TiN interface. Pore walls perpendicular to the plane of the FIB cut are thicker in the resulting sample and therefore appear brighter in the dark-field image than those parallel to the plane of the FIB cut. Pores likely develop when the surrounding Cu_3_Si hillocks break through the Cu layer, thereby partly lifting it off of the Si(001) substrate as a result of 150% volume expansion associated with Cu_3_Si formation (Fig. 4(b))^30^.

EDX maps (not shown) reveal that the bright dots at the TiN/Si interface (indicated by the dashed white rectangle in Fig. 4(b)) are composed of Cu and Si, representing an early stage in the interfacial reaction leading to formation of Cu_3_Si hillocks.

Figure 4(c) is an STEM image of a Cu/TiN bilayer annealed at 900 °C. The TiN layer is bent and barrier breakdown has occurred at the column boundaries. Diffused Cu has reacted to form Cu_3_Si, visible as the bright phase beneath the barrier layer. Figure 4(d) is a higher-resolution STEM image of the same sample with a corresponding EDX line profile acquired along the red arrow shown in Fig. 4(e). The presence of Si on top of, and Cu beneath, the TiN barrier is a clear indication of barrier failure. EDX line scans over the cross-section of the failed TiN barrier shown in Fig. 4(e) reveal alternating Ti-rich regions within columns and Cu-rich regions at the column boundaries. These results, together with the finding of intergranular porosity in the XTEM image of the as-deposited bilayer (Fig. 4(a)), establish that the column boundaries act as the initial fast diffusion paths during annealing. Correlated increases in Cu and Si EDX signal intensities indicate the formation of Cu silicide as a TiN/Si interfacial reaction product after Cu diffusion through the barrier, in good agreement with the results and discussion in the preceding section. The Cu_3_Si compound melts at *T*_*m*_ = 859 °C and will re-solidify in the cooling period of the 900 °C annealing treatment, thereby forming a seam around the disintegrated TiN columns and essentially sintering them together as visible in Fig. 4(c,d). As a result, the sheet resistance drops from ~14 Ω/□ for the buckled, partially reacted 700 and 800 °C annealed Cu/TiN bilayers to ~1.8 Ω/□ for the 900 °C annealed fully-reacted Cu/TiN bilayer as displayed in Fig. 3.

**Figure 5:**
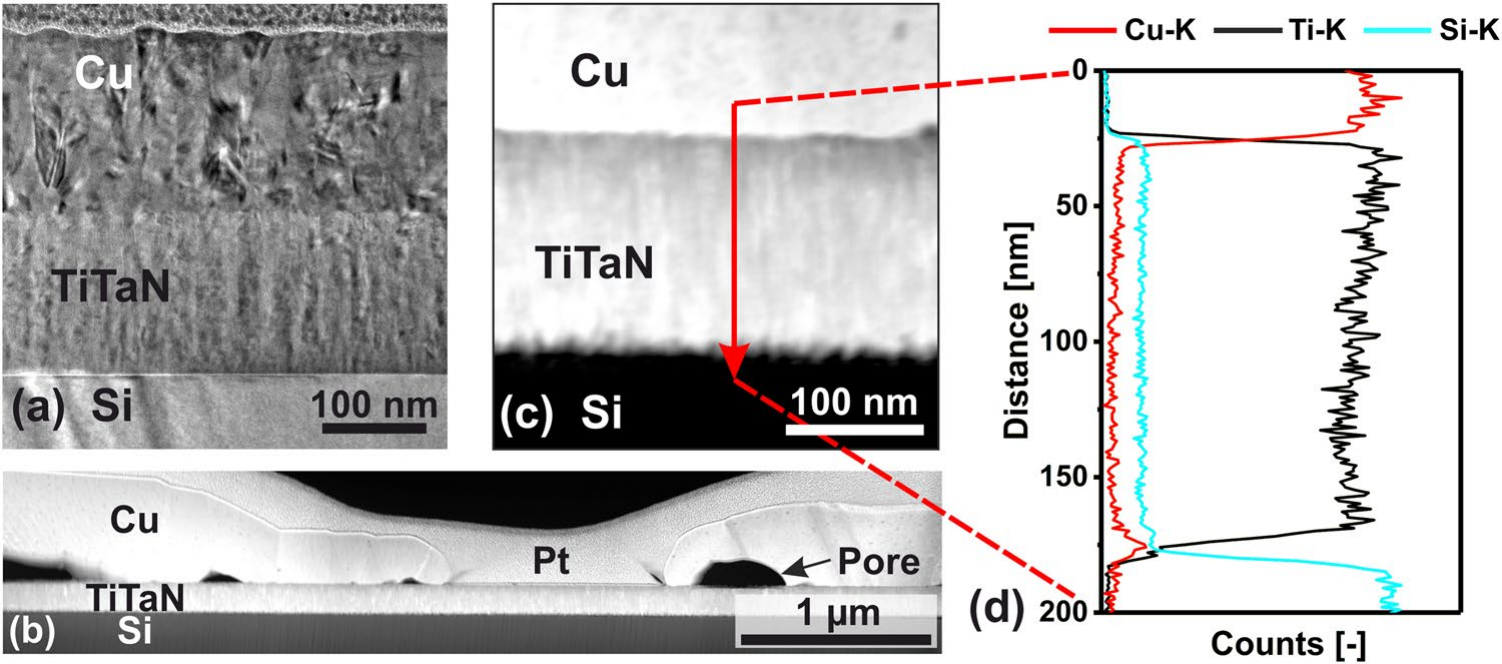
Cross-sectional transmission electron micrographs of a Cu/Ti_0.84_Ta_0.16_N bilayer grown on Si(001). (**a**) bright-field XTEM image of the as-deposited bilayer, (**b**) *Z*-contrast STEM overview of the bilayer after annealing at 900 °C, and (**c**) a higher-magnification STEM image of the same 900 °C annealed bilayer with (**d**) a corresponding EDX line scan acquired along the red arrow in (**c**).

XTEM analyses of annealed Cu/Ti_0.84_Ta_0.16_N bilayers are summarized in Fig. 5. As-deposited Ti_0.84_Ta_0.16_N barriers, Fig. 5(a), exhibit a columnar structure (<*d>* ~110 Å) with dense boundaries. Densification is facilitated by pulsed Ta ion irradiation during which momentum transfer promotes atomic intermixing in the near-surface region of the growing film^16^. The Cu layer consists of larger columns (<*d>* ~350 Å) with fewer voids than Cu films deposited on TiN. No changes are apparent in the barrier layer after annealing at 700 °C; we observe Cu grain growth to an average column diameter <*d>* of ~5000 Å and the formation of pores, ~500 Å in diameter, at the Cu/barrier interface due to the initiation of solid-state dewetting. Cu dewetting increases during annealing at 900 °C, leaving µm-sized regions of the Ti_0.84_Ta_0.16_N surface Cu-free as shown in the *Z*-contrast STEM image in Fig. 5(b). This is interpreted as a sign of high diffusion barrier effectiveness; the reduced wettability of Cu on the transition-metal nitride at elevated temperatures suggests that no detectable interface reaction or interdiffusion has occurred. Indeed, the large-scale integrity of the 900 °C annealed Ti_0.84_Ta_0.16_N film is observable in the *Z*-contrast STEM overview image in Fig. 5(b). At higher resolution (Fig. 5(c)), Ti_0.84_Ta_0.16_N/Si interface roughening is visible, indicating initial barrier degradation during the annealing treatment. However, no interdiffusion reaction zones are evident.

Figure 5(d) is an EDX line profile obtained along the direction of the arrow in Fig. 5(c). The Cu/Ti_0.84_Ta_0.16_N interface exhibits a rapid decrease in the intensity of the Cu-L edge, while the intensity of the Ti-K edge increases over a relatively sharp, ~10-nm-wide, interface with no indication of interdiffusion. The elevated Si intensity in the Ti_0.84_Ta_0.16_N layer is an artefact arising from the energy overlap of the Si-K and the Ta-M edges. The Ti_0.84_Ta_0.16_N/Si interface is approximately 16 nm wide as determined from the EDX profile, and not as sharp as the Cu/Ti_0.84_Ta_0.16_N interface, as a result of the initiation of the interfacial reaction, ultimately leading to formation of Cu_3_Si. Additionally, a pronounced increase in Cu intensity is apparent at the interface, in agreement with XRD results showing the presence of Cu_3_Si after 900 °C annealing (Fig. 2(b)) and consistent with the rough appearance of the barrier/substrate interface in Fig. 5(c).

Overall, the Cu diffusion through the DCMS/HiPIMS Ti_0.84_Ta_0.16_N barrier during annealing at 900 °C is significantly decreased compared to the DCMS TiN barrier. In the latter case, all Cu is consumed to form Cu_3_Si, while in the former case, the Cu metallization is still present, albeit in an agglomerated dewetted form, on the Ti_0.84_Ta_0.16_N layer and EDX results show that Cu_3_Si is only formed at the barrier/substrate interface. Thus, there is only minor Cu diffusion through the dense Ti_0.84_Ta_0.16_N barrier during 1-h 900 °C annealing. In contrast, the TiN barrier already loses its structural integrity after annealing at ≤700 °C, due to pronounced Cu diffusion and the formation of large Cu_3_Si hillocks.

## Conclusions

We present a comparison of the performance of DCMS TiN and DCMS/HiPIMS Ti_0.84_Ta_0.16_N diffusion barriers between Cu and Si(001), both deposited with no substrate heating in order to comply with increasingly low thermal budgets in the semiconductor industry.

Cu/barrier/Si(001) stacks, as-deposited as well as vacuum annealed at 700, 800, and 900 °C for 1 h, are investigated by scanning electron microscopy, X-ray diffraction, sheet resistance measurements, and cross-sectional transmission electron microscopy in conjunction with energy-dispersive X-ray spectroscopy to evaluate interlayer diffusion. The TiN barrier exhibits extensive interdiffusion after annealing for 1 h at ≤700 °C. Cu diffuses through the TiN barrier along fast diffusion paths at open column boundaries to the Si(001) substrate as observed by XTEM. Cu_3_Si nucleates at the TiN/Si(001) interface as an interdiffusion reaction product. During annealing at 900 °C, the silicide proceeds to grow until the entire Cu layer is consumed. This leads to structural breakdown of the columnar TiN barrier. In contrast, only minor Cu diffusion is evident in the stable Ti_0.84_Ta_0.16_N diffusion barrier even after annealing at 900 °C. That is, the Ti_0.84_Ta_0.16_N barrier remains structurally intact at 900 °C. This improved performance is attributed to barrier densification by pulsed bombardment with energetic, heavy Ta ions inherent to the hybrid DCMS/HiPIMS deposition.

## Methods

### Thin film deposition

Cu/TiN and Cu/Ti_0.84_Ta_0.16_N bilayers are deposited on Si(001) substrates in a CC800/9 CemeCon magnetron sputtering system with a base pressure of 5 × 10^−4^ Pa (3.7 × 10^−6^ Torr). A detailed description of the deposition system is presented in ref.^16^. The Si(001) substrates are B-doped and have a resistivity between 1 and 20 Ω-cm. Single layer 2000-Å-thick DCMS TiN and 1500-Å-thick DCMS/HiPIMS Ti_0.84_Ta_0.16_N reference samples are deposited on Si(001) substrates without Cu overlayers.

The deposition system contains cast rectangular 8.8 × 50 cm^2^ Ti, Ta, and Cu targets with a target-to-substrate separation of 18 cm. Si(001) substrates (2 × 1 cm^2^) with a native SiO_2_ layer are cleaned in ultrasonic baths of acetone and isopropyl alcohol and blown dry with dry N_2_ immediately prior to loading them into the vacuum system. Prior to deposition, each target is separately sputter etched in pure Ar (flow rate = 650 sccm) for 1 min, with shutters protecting the substrates and adjacent targets. Sputter etching is carried out in DCMS mode, with the target power set to 5, 3, and 2 kW for Ti, Ta, and Cu, respectively. Subsequently, the transition-metal nitride layers are deposited in mixed Ar/N_2_ atmospheres with the Ar flow set to 350 sccm; the N_2_ flow is automatically regulated by a feedback loop to maintain a constant total deposition pressure of 0.42 Pa (3 mTorr). In these experiments, the maximum substrate temperature due to plasma heating is 90 °C during TiN and TiTaN film growth^16^.

For DCMS TiN deposition, the Ti target power is 6 kW and the Si substrates are electrically floating at a potential *V*_*f*_ = −10 V, resulting in a TiN deposition rate of 330 Å/min and a total TiN film thickness of 2000 Å. The deposition conditions are the same for DCMS/HiPIMS Ti_0.84_Ta_0.16_N, except that the Ta target is operated in HiPIMS mode with an average power of 1.5 kW (average target power density per period: 3.4 W/cm^2^, average target power density per pulse: 170 W/cm^2^) at a pulsing frequency of 100 Hz (2% duty cycle). A negative substrate bias of 60 V is applied synchronously with the metal-ion-rich portion of each 200-µs-long HiPIMS pulse, beginning at 40 µs after pulse initiation and ending at 100 µs. Mass spectrometry measurements carried out with the spectrometer orifice at the substrate position are described in detail in refs^16,37,38^. Between HiPIMS pulses, the substrates are at floating potential and the amount of deposited TiN is <2.2 × 10^−3^ monolayers. The net DCMS/HiPIMS TiTaN deposition rate is 340 Å/min with a total TiTaN film thickness of 1500 Å. The Ta/(Ti + Ta) fraction is 0.16, as determined by EDX measurements carried out on fracture cross-sections. N/(Ti + Ta) fractions are 1.00 ± 0.03, based upon Rutherford backscattering spectroscopy using a 2.0 MeV ^4^He^+^ probe beam incident at 10° with respect to the surface normal and detected at a 172° scattering angle as described in ref.^15^.

Following nitride diffusion barrier growth, the substrate holder is rotated to face the Cu target. Without breaking vacuum, a 1600-Å-thick Cu layer is deposited in pure Ar at a pressure of 0.42 Pa (3 mTorr, corresponding Ar flow rate = 440 sccm) on the transition-metal nitride films at a constant target power of 2 kW and a substrate bias of −100 V. The Cu deposition rate is 1500 Å/min. The Cu, DCMS TiN, and DCMS/HiPIMS Ti_0.84_Ta_0.16_N layer thicknesses are determined from XTEM analyses.

### Annealing

The Cu/transition-metal nitride bilayers on Si(001) are isothermally annealed in an HTM-Reetz vacuum annealing furnace with a base pressure of 10^−5^ Pa (7.5 × 10^−8^ Torr). The bilayers are heated at 30 °C/min to annealing temperatures *T*_*a*_ of 700, 800, and 900 °C, held at *T*_*a*_ for 1 h, and passively cooled from *T*_*a*_ to room temperature. The cooling rate decreases from ~20 °C/min at temperatures near *T*_*a*_ to ~3 °C/min near room temperature.

### Thin film characterization

Plan-view SEM micrographs before and after annealing are obtained in a Zeiss Auriga SMT SEM equipped with secondary-electron and backscattered-electron detectors. Average surface roughnesses *R*_*a*_ of the unannealed Cu layers are determined over areas of 0.5 × 0.5 mm^2^ at the sample centre using a Wyko NT 1000 optical three-dimensional white-light profiling system. XRD patterns from as-deposited and annealed bilayer samples are obtained using a Bruker-AXS D8 Advance diffractometer operated with Cu-K_α_ radiation, parallelized by a primary Göbel mirror, at 2° grazing incidence. The diffractograms are recorded using an energy-dispersive SolX detector with a 0.12° soller slit collimator.

Sheet resistances *R*_*S*_ of as-deposited and annealed Cu layers, as well as un-metallized DCMS TiN and DCMS/HiPIMS Ti_0.84_Ta_0.16_N layers, are determined with a linear-array Jandel four-point probe (1.00 mm probe spacing) based on measurements at three randomly chosen positions near the centre of each sample. The output current *I* of the probe is set to 9.990 mA DC, and the sheet resistance *R*_*s*_ is calculated from the measured voltage *V* according to the following formula

1 $${R}_{S}=\frac{\pi }{ln2}\frac{V}{I}.$$

Since the sheet resistivity of as-deposited Cu is two orders of magnitude lower than the resistivity of the underlying nitride films and six orders of magnitude lower than that of the Si substrate, we assume that when testing Cu/nitride bilayer samples any changes in sheet resistivity, and thus, sheet resistance, can be primarily related to microstructural changes in the Cu layer.

XTEM samples are prepared in an Orsay Physics Cobra Z-05 FIB unit equipped with a Ga^+^ source. The final thinning step is carried out with a 5 kV Ga^+^ beam to minimize Ga^+^ implantation. Microstructural and analytical XTEM investigations are performed in a FEI Tecnai G^2^ TF20 UT TEM equipped with a field-emission source operated at 200 kV. Atomic-number *Z*-contrast scanning transmission electron microscopy utilizing a high-angle annular dark-field detector is employed in combination with EDX line profiling (1 data-point/10 Å) to determine interdiffusion in annealed samples.

### Data availability

The datasets generated during the current study are available from the corresponding author upon reasonable request.

## Electronic supplementary material

Supplementary Information
