# Supplementary material for: Enhanced Ti0.84Ta0.16N diffusion barriers, grown by a hybrid sputtering technique with no substrate heating, between Si(001) wafers and Cu overlayers
Source: Sci Rep. 2018 Mar 29;8:5360. doi: 10.1038/s41598-018-23782-9 (PMC5876326; doi:10.1038/s41598-018-23782-9)
Supplement: Supplementary file 1 — Supplementary Information [file 41598_2018_23782_MOESM1_ESM.pdf]

Supplementary Information

Enhanced $Ti_{0.84}Ta_{0.16}N$ diffusion barriers, grown by a hybrid sputtering technique with no substrate heating, between Si(001) wafers and Cu overlayers

Marlene Mühlbacher[1,2], Grzegorz Greczynski[2], Bernhard Sartory[3], Nina Schalk[1], Jun Lu[2], Ivan Petrov[2,4], J. E. Greene[2,4], Lars Hultman[2], Christian Mitterer[1]

[1] Department of Physical Metallurgy and Materials Testing, Montanuniversität Leoben, Franz-Josef-Strasse 18, A-8700 Leoben, Austria

[2] Thin Film Physics Division, Department of Physics, Chemistry, and Biology (IFM), Linköping University, S-581 83 Linköping, Sweden

[3] Materials Center Leoben Forschung GmbH, Roseggerstrasse 12, A-8700 Leoben, Austria

[4] Department of Materials Science, Physics, and the Frederick Seitz Materials Research Laboratory, University of Illinois, Urbana, Illinois 61801, USA

Determination of residual stresses

Residual stresses in the nitride layers are determined with the $sin^2\Psi$ method in Seemann-Bohlin geometry with a grazing incidence angle of 2°. In as-deposited state, the Ti(Ta)N (200) reflection is excluded from the measurement, since its vicinity to the Cu (111) reflection does not allow for a successful Gaussian peak fit.

The residual stress σ as a function of the film's Young's modulus *E* is calculated as

$$\sigma = k * \frac{1}{a_0} * \frac{E}{1+\nu} \ ,$$

with *k* as the slope of the $sin^2\Psi$ plot and a_0 as the strain-free lattice parameter, where $sin^2\Psi = \frac{2\nu}{1+\nu}$. A Poisson ratio $\nu = 0.3$ is assumed for the calculations.

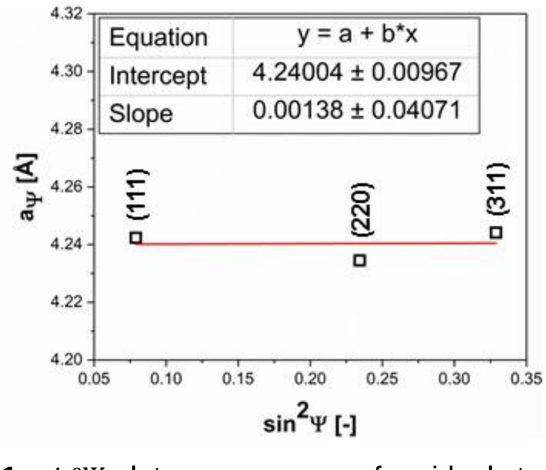

Supplementary Figure S1. $sin^2\Psi$ plot as a measure of residual stresses in the TiN layer in the as-deposited Cu/TiN bilayer sample. The as-deposited TiN film is essentially stress free, with $\sigma = 0.0003 \times E$.

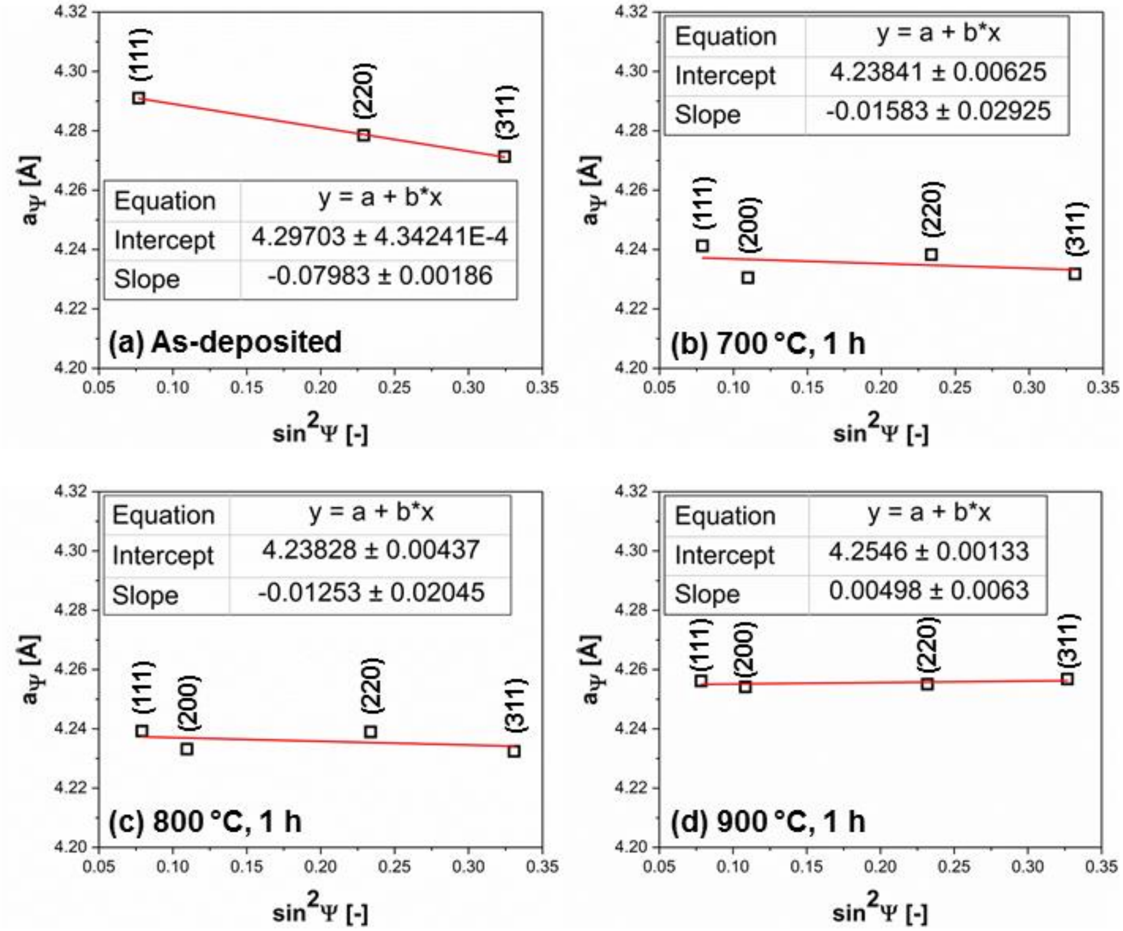

Supplementary Figure S2. *sin*²Ψ plot as a measure of residual stresses in the $Ti_{0.84}Ta_{0.16}N$ layer in the (a) as-deposited, (b) 700 °C annealed, and (c) 800 °C annealed Cu/$Ti_{0.84}Ta_{0.16}N$ bilayer samples as well as the (d) 900 °C annealed single layer $Ti_{0.84}Ta_{0.16}N$ sample. To exclude the influence of any diffusion reaction products on the measured residual stress, the measurement for the 900 °C annealed sample was carried out on single layer $Ti_{0.84}Ta_{0.16}N$.

The as-deposited $Ti_{0.84}Ta_{0.16}N$ film is under compressive stress σ = -0.0144×*E.* Annealed films are stress free, with the 900 °C annealed sample under tensile stress σ = 0.0009×*E.*

Supplementary Table S3. Evolution of the sheet resistances R_S and sheets resistivities ρ_S of Cu/TiN and Cu/$Ti_{0.84}Ta_{0.16}N$ bilayers and TiN and $Ti_{0.84}Ta_{0.16}N$ single layers as a function of annealing temperature T_a. The sheet resistances/resistivities after 900 °C annealing correspond to fully-reacted (Cu/TiN) and dewetted (Cu/$Ti_{0.84}Ta_{0.16}N$) bilayers.

	**as-deposited**		**700 °C**		**900 °C**	
	**R_S [Ω/□]**	**ρ_S [µΩ-cm]**	**R_S [Ω/□]**	**ρ_S [µΩ-cm]**	**R_S [Ω/□]**	**ρ_S [µΩ-cm]**
**Cu/TiN**	1.06±0.09	17.0±1.5	13.80±0.46	221.0±7.2	1.83±0.05	36.6±1.0
**single layer TiN**	42.22±1.01	844.3±20.6	38.79±0.70	776.0±13.9	2.59±0.14	51.7±2.9
**Cu/$Ti_{0.84}Ta_{0.16}N$**	0.31±0.05	4.9±0.8	0.14±0.01	2.3±0.1	2.06±0.01	30.9±0.1
**single layer $Ti_{0.84}Ta_{0.16}N$**	7.70±0.15	115.4±2.1	7.60±0.11	114.2±1.5	4.97±0.49	74.5±7.3
